# A study on the effect of nutrition education based on the goal attainment theory on oral nutritional supplementation after colorectal cancer surgery

**DOI:** 10.1007/s00520-023-07905-1

**Published:** 2023-07-06

**Authors:** Jun-fang Pi, Jing Zhou, Ling-ling Lu, Lan Li, Chen-rong Mao, Ling Jiang

**Affiliations:** Department of Gastrointestinal Surgical Ward, The Affiliated Suzhou Hospital of Nanjing Medical University, Suzhou Municipal Hospital, No. 26 of Daoqian Street, Gusu District, Suzhou, 215000 China

**Keywords:** Colorectal cancer, Oral nutritional supplementation, Goal Attainment Theory, Nutrition education, Health education, Nutritional status

## Abstract

**Objective:**

To investigate their compliance with postoperative oral nutritional supplementation and nutritional outcomes.

**Methods:**

A total of 84 patients with colorectal cancer surgery with NRS-2002 risk score ≥ 3 who were treated with oral nutritional supplementation were selected and divided into control and observation groups according to the random number table method, with 42 cases in each group. The control group received conventional oral nutritional supplementation and dietary nutrition education; the observation group established a nutrition intervention group based on the Goal Attainment Theory and carried out individualized nutrition education based on the Goal Attainment Theory. The nutritional indicators at 1 day postoperative, 7 days postoperative, oral nutritional supplementation adherence scores at 7 and 14 days postoperative, and the attainment rate of trans-oral nutritional intake at 21 days postoperative were compared between the 2 groups of patients.

**Results:**

There was no statistically significant difference between the nutritional status indexes of the 2 groups of patients before the intervention, *p* > 0.05; when comparing the prealbumin of the 2 groups of patients at 7 days postoperatively, the prealbumin level of the patients in the observation group at 7 days postoperatively (200.25 ± 53.25) was better than that of the control group (165.73 ± 43.00), with a *p* value of 0.002, and the difference was statistically significant (*p* < 0.05). Comparison of oral nutritional supplementation adherence scores at 7 and 14 days postoperatively showed that ONS treatment adherence scores were better than those of the control group, with statistically significant differences (*p* < 0.05). When comparing the attainment rate of oral nutritional intake at 21 days after surgery, the difference was statistically significant (*p* < 0.05).

**Conclusion:**

Nutritional education based on the Goal Attainment Theory can effectively improve the adherence to oral nutritional supplementation therapy and protein intake attainment rate of colorectal cancer patients after surgery and effectively improve the nutritional status of patients.

## Introduction

Colorectal cancer (CRC) is one of the most common gastrointestinal tumors, ranking 3rd in incidence and 4th in mortality among malignant tumors worldwide [1]. The widespread introduction of the Enhanced Recovery After Surgery (ERAS) program includes a series of evidence-based multimodal interventions aimed at accelerating early postoperative recovery [2]. Oral food intake as early as possible is a key intervention of the ERAS program. However, current ERAS guidance either does not include a specific recommendation for volume of postoperative oral fluids/foodstuffs or suggests ad-lib fluids [2]. In the case of inadequate coverage of the calorie and protein requirement, perioperative nutrition therapy strives for supplementing the primarily oral diet by oral nutritional supplements (ONS) and enteral nutrition [3]. The European Society for Clinical Nutrition and Metabolism guidelines [4] in 2017 suggested that patients at nutritional risk or with malnutrition should all receive nutrition education and nutrition support five-step therapy [5]. The KEPAN consensus report on oral nutritional supplements (ONS) [6] recommends ONS as the best mode of perioperative nutritional support, and early resumption of oral feeding and early implementation of ONS reduces the incidence of complications, especially infectious complications. ONS is more conducive to the restoration of intestinal flora and immune function in patients, which can improve clinical outcomes and is cost-effective, but its therapeutic effect is related to patient adherence. A study showed that only 24.04% of the total number of patients had high adherence of ONS intake (correct method of configuration, temperature, number of doses, single oral dose, and standard daily energy intake of ONS) in the early postoperative period [7]. ONS-related guidelines [6, 8] recommend that nutrition education should be enhanced in the implementation of ONS, and it is crucial for the successful implementation of ONS to educate patients about nutrition, but there is less literature on the specific implementation methods of nutrition education.

Imogene M. King’s Theory of Goal Attainment [9] focuses on the role of nurse-patient interactions to encourage patients to take the initiative and achieve optimal health. If the nurse-patient relationship is perceived to be accurate, it will facilitate communication, which in turn will facilitate the achievement of common goals, thereby improving patient adherence and confidence. Currently, the goal attainment theory is mainly focused on the application of nursing practice and education of patients with chronic diseases [10–12]. The implementation of education based on the goal attainment theory is an approach to the nursing process that includes four parts: interactive assessment, common goal attainment plan, implementation measures, and evaluation of effectiveness, highlighting the concept of holistic, personalized, and humanized care. In view of this, this study implemented nutrition education based on the goal attainment theory for postoperative colorectal cancer patients to stimulate the interest of patients and family members in learning nutrition knowledge, and nurse and patients jointly formulated ONS and diet plans and participated in the perioperative nutrition management of patients, in order to improve patients’ adherence to ONS nutrition support, improve patients’ nutritional status, and promote patients’ postoperative recovery, which is reported as follows.

## Materials and methods

### General information

Patients with colorectal cancer surgery treated with ONS in the gastrointestinal surgery department of a tertiary care hospital from May 2022 to December 2022 were selected for this study. Inclusion criteria are as follows: ① pathological diagnosis of colon and rectal cancer; ② underwent surgical treatment for colon and rectal cancer; ③ preoperative nutritional risk screening NRS2002 score ≥ 3; ④ underwent oral nutritional supplementation nutritional intervention; ⑤ had certain reading and comprehension ability, could communicate normally with language, and voluntarily cooperated with the survey. Exclusion criteria are as follows: ① combined with moderate to severe cardiac, hepatic, pulmonary, renal and other visceral diseases; ② serious postoperative infections, intestinal fistulas, and other complications. Rejection criteria are as follows: those with incomplete questionnaire assessment information during the intervention. Prior to the conduct of this study, informed consent was obtained from patients to participate in this study, and the study passed the ethical review of the hospital ethics committee, with ethics review number K-2021–050-K01. This study was a randomized controlled interventional quantitative study with single-blind, randomized groups of subjects and simple convenience sampling. The excel sheet RANDBETWEEN function was used to generate a table of random numbers, the grouping scheme was placed in an opaque sealed envelope, patients meeting the inclusion criteria were given numbers, the corresponding numbered envelopes were opened, and the intervention was performed according to the grouping scheme inside the envelope.

The sample size formula [13] was used for the comparison of the two overall means: *n*1 = *n*2 = 2[(*μα* + *μβ*)*σ*/*δ*]2, with settings of bilateral *α* = 0.05 and 1 − *β* = 0.90, and equal numbers in the control and observation groups. The literature was reviewed [14], and the mean and standard deviation were obtained for the observation and control groups, and the oral supplementation adherence score was (22.5 ± 4.8) for the observation group and (19.26 ± 3.053) for the control group, and the sample size was calculated. After excluding one case of postoperative occurrence of mammary fistula and rejecting one case of incomplete information, valid data were obtained for a total of 84 cases, 42 in the control and intervention groups, respectively.

### Methods

#### Control group: ONS nutritional support + conventional nutrition education

##### ONS nutritional support

The ONS preparation is either whole protein enteral nutrition powder Nutrison (produced by Newdishia, with 320 g in a can, totaling 1940 kcal energy, every 100 g contains 18.5 g of protein, 56.4 g of carbohydrates, 18.2 g of fat, multiple vitamins, and trace elements) or Ensure (produced by Abbott, with 400 g in a can, totaling 1800 kcal energy, every 100 g contains 15.8 g of protein, 59.6 g of carbohydrates, 15.8 g of fat, multiple vitamins, and trace elements).

##### Intervention method

One to 3 spoons of Ensure or 3 to 5 spoons of Nutrison with 50 to 100 ml of warm boiled water (37 to 40 degrees) were taken 2–3 h after surgery, with a gradual increase from less to more, from thin to thick, and 6 spoons plus 200 ml of Ensure or 9 spoons of Nutrison with 200 ml of warm water taken between meals, with 2 to 3 times daily, and the “3 + 3 diet pattern” of three regular meals + three ONS could be used [15], with an energy intake of 400 to 600 kcal/day of ONS. The postoperative ONS nutritional support intervention was performed until 14 days postoperatively.

The demand for parenteral nutrition was calculated based on the recommendations of the European Society for Clinical Nutrition and Metabolism (ESPEN) guidelines, which recommend the use of parenteral nutrition for patients who cannot meet oral/enteral energy requirements within 5–7 days after surgery. The recommended intake of protein is 1.5 g/kg body weight [16].

##### Conventional nutrition education

The primary nurse distributed ONS preparations as prescribed by the physician and provided ONS-related nutrition education to patients and families. The form of education included verbal preaching and distribution of a self-created paper version of the Oral Nutritional Supplementation Preaching Manual, developed by the investigator based on the guideline literature [8]. The content of nutrition education is as follows: hospital basic diet (semi-volume fluid, fluid, semi-liquid) of food types, meal frequency, the amount of meals, to follow a high-calorie, high-protein, high-vitamin diet, nutritionally balanced, divided into multiple meals, chew and swallow diet principles; ONS preparations of the nutritional composition, the advantages of ONS, ONS brewing methods and precautions, the number of ONS meals and the observation and treatment of adverse reactions; to guide patients to develop an activity plan to gradually increase the amount of activity. Patients were followed up by telephone after discharge to find out the type of diet, number of meals, ONS consumption, weight, and whether they had any physical symptoms.

#### Observation group: ONS nutritional support program was the same as the control group

Quality control of nutrition education based on the goal attainment theory.

Nutrition Support Team (NST) was established, and NST team members were designated as the special person in charge of patient nutrition support. On the basis of routine nutrition education by primary nurses, NST team members carried out professional nutrition education, consultation, supervision, and management based on the goal attainment theory. After discharge, the patients joined the medical nurse-patient communication QQ group “nutrition education group” on the basis of the telephone follow-up of the control group for nutrition consultation and interaction. The timing of communication between members of the nutrition support group and patients and families: 1 to 2 days after admission, 1 to 2 days after surgery, 7 days after surgery, and 1 to 2 days before discharge, with each communication lasting 10–15 min [17]. Staff training was conducted before the study: (i) to understand the design of the study and to master the observation index collection method, intervention process, and precautions; (ii) to learn the knowledge about the King’s Theory of Goal Attainment and the process and method of nutrition promotion based on the theory; (iii) to control the quality of the promotion by the nurse leader and the department nurse leader.

##### A nutrition promotion approach based on the Goal Attainment Theory

Nutrition education based on goal attainment theory includes four steps: interactive assessment, goal attainment plan, implementation measures, and evaluation of effectiveness. After understanding the patient’s disease, education level, body mass index, nutrition risk score, and nutrition support pathway through medical records, the nutrition education nurse communicated with the patient and family members at the bedside to understand their degree of mastery of nutrition-related knowledge (cognition of ONS, dietary principles), comprehension, personal eating habits and tastes, appetite status, whether there is nausea and vomiting and other physical discomfort or weight loss, assess the patient’s existence problems, formulate health education plans on demand for patients’ different age characteristics, cognitive status, preferred nutrition education methods, etc., and select suitable enteral nutrition preparations and brewing methods according to patients’ favorite tastes and intestinal adaptations. They instructed patients to use multimedia to watch the nutrition science video made and recorded by nutrition specialist nurses after reviewing literature. The video was reviewed by doctors, nurse managers, and dietitians, and the science video introduced the physiological functions of the stomach and intestines, the effects of surgery on the body, the risks and consequences of malnutrition, the principles and methods of the five-step treatment of nutritional support, the common misconceptions about the diet of tumor patients, the principles and precautions of postoperative diet, what the high-quality high-protein foods are, the dietary precautions for stoma patients, the advantages of oral nutritional supplementation therapy, the method and precautions of ONS brewing, and the indicators of nutritional monitoring in easy-to-understand language. They helped patients and family members understand the importance of nutritional risk assessment and monitoring of nutritional indicators, helped patients and family members gradually develop correct eating habits and dietary principles, the method and frequency of ONS supplementation, and were able to adhere to ONS treatment, with additional ONS supplementation of 400–600 kcal per day in addition to diet. Specialized nurses supervise the patients’ eating, the administration of oral nutritional supplements, and follow up on the patients’ nutritional indicators for effect evaluation. Patients’ dietary types and reasons for substandard caloric intake of ONS were analyzed, and those with abdominal pain and bloating discomfort during enteral nutrition were assessed for enteral nutrition intolerance using the enteral nutrition tolerance scale [18], while patients were encouraged to elaborate on individual tolerance of nutritional preparations and coping methods; the attainment interactive nursing measures were revised and adjusted, and the process of attainment interaction was re-entered to reinforce nutrition education; the nursing intervention was terminated for those who met the standard. Before discharge, patients or their families joined the “nutrition education group” medical care and patient communication group through QQ and promptly interacted with doctors and clinical nutrition nurses in the “nutrition education group” when they encountered problems. Patients were encouraged to improve their self-monitoring ability, do daily weight monitoring, learn to calculate the amount of calories and protein intake of food and the energy intake of ONS, and record it on the ONS implementation table.

### Research tools

#### Medication adherence questionnaire

The medication adherence questionnaire, revised by Yunxia Zhu [14] on the basis of the Morisky Medication Adherence Scale, was used to evaluate oral nutritional supplement adherence, with six items, intra-group correlation coefficients of 0.80–1.00 for each item and total questionnaire score, and a Cronbach coefficient of 0.88 for questionnaire reliability analysis. On postoperative day 7 and 14, patients were asked about the frequency of “taking oral nutritional supplements as often as prescribed,” “taking them as long as prescribed,” “taking them at the concentration prescribed,” and “forgetting to take them” during 1 week of oral supplementation, and each item was scored on a 5-point Likert scale up to 30 points. Item 6, Forgetting to take medication, is a reverse item. The higher the total score, the better the medication adherence. Patients in the hospital were surveyed by face-to-face questionnaires; patients discharged from the hospital were asked to fill in the questionnaires on the researcher’s behalf by telephone follow-up.

#### Self-made dietary, oral nutritional supplementation record sheet

A homemade form was used to record the time and amount of oral ONS taken by the patient each day and to calculate the daily caloric intake of the patient through oral nutritional supplementation after surgery. Nutritional support guidelines for oncology patients [19, 20] recommend that the target protein requirement for patients is 1.0–2.0 g/kg day, and the minimum requirement is 1.0 g/kg day. The amount of protein consumed by patients’ diet and the amount of protein consumed by ONS were calculated with the Mint Dietitian APP, and reaching the minimum requirement of 1.0 g/kg day was considered as meeting the standard, and the 21-day postoperative protein intake attainment rate was calculated for both groups.

### Observed indicators

The nutritional indicators such as serum albumin value, prealbumin value, and hemoglobin value at 1 and 7 days postoperatively were compared between the two groups; the ONS intake compliance scores at 7 and 14 days postoperatively were compared between the two groups; and the oral nutritional protein intake compliance rate at 21 days postoperatively was compared between the two groups.

### Statistical methods

Excel 2013 was used for data entry and double-checking; the statistical software SPSS 25.0 was used to import data for data analysis. The measurement data were expressed as mean ± standard deviation (x ® ± s), and two groups were compared using independent samples *t* test if they conformed to normal distribution; otherwise, the non-parametric test rank sum test was used; categorical data were expressed as counts, percentages (*n*, %), and all theoretical frequencies *T* ≥ 5 and total sample size *n* ≥ 40 were tested by Pearson’s chi-square, and if theoretical frequencies *T* < 5 but *T* ≥ 1 and *n* ≥ 40, a test of continuity-corrected chi-square was used. The difference was considered statistically significant at *p* < 0.05.

## Results

### Comparison of general information between the two groups of patients

General data such as age, gender, surgical procedure, education level, and preoperative BMI were compared between the two groups, and it was determined that the differences in general data after enrollment were not statistically significant (*p* > 0.05) to ensure comparability of the subsequent observations between the two groups, as shown in Table 1.

**Table 1:** Comparison of general information between the two groups of patients

Items	Experimental group (*n* = 42)	Control group (*n* = 42)	*T* value/χ^2^ value	*p* value
Age (years) $\overline{x }$ ± *s*	66.55 ± 1.84	66.00 ± 2.16	− 1.93	0.847
Gender (*n*, %)				
Female	15 (35.7%)	15 (35.7%)	0.000	1.000
Male	27 (64.3%)	27 (64.3%)		
Surgery method (*n*, %)				
Colon surgery	23 (54.8%)	24 (57.1%)	0.48	0.826
Rectal surgery	19 (45.2%)	18 (42.9%)		
Educational level (*n*, %)				
Junior high school and below	31 (73.8%)	25 (59.5%)	1.929	0.165
High School and above	11 (26.2%)	17 (40.5%)		
Pre-operative BMI (kg/m^2^) $\overline{x }$ ± s	22.39 ± 3.46	23.09 ± 2.75	1.038	0.302

### Comparison of preoperative nutritional status between two groups of patients

The preoperative nutritional status of the two groups was compared, and it was found that there was no statistically significant difference in preoperative albumin, prealbumin, and hemoglobin between the observation group and the control group (*p* > 0.05), further ensuring the comparability of the two groups’ observations, as shown in Table 2.

**Table 2:** Comparison of preoperative nutritional status between two groups of patients ($\overline{\mathrm{x} }$ ± s)

Group	Number of cases	Albumin (g/L)	Prealbumin (mg/L)	Hemoglobin (g/L)
Observation group	42	38.25 ± 4.63	205.65 ± 63.29	119.62 ± 24.94
Control group	42	38.66 ± 5.00	199.68 ± 63.16	121.19 ± 20.10
*t-value*		0.394	0.431	0.318
*p-value*		0.694	0.681	0.751

### Comparison of the nutritional status of the two groups of patients 1 day after surgery and 7 days after surgery

A comparison of the nutritional status of patients in the two groups on postoperative day 1 and postoperative day 7 revealed that the nutritional status of patients in the observation group on postoperative day 1 was not statistically significant compared with that of the control group. However, the prealbumin was significantly higher in the observation group than in the control group 7 days after surgery, and the difference was statistically significant (*t* value − 3.268, *p* < 0.05), as shown in Table 3.

**Table 3:** Comparison of the nutritional status of patients in the two groups on postoperative day 1 and postoperative day 7 ($\overline{\mathrm{x} }$ ± s)

Group	Number of cases (*n*)	1 day postoperative			7 days postoperative		
		Albumin (g/L)	Prealbumin (mg/L)	Hemoglobin (g/L)	Albumin (g/L)	Prealbumin (mg/L)	Hemoglobin (g/L)
Observation group	42	35.42 ± 4.84	154.57 ± 45.49	111.33 ± 15.18	39.48 ± 4.02	200.25 ± 53.25	115.83 ± 14.75
Control group	42	34.99 ± 5.04	156.80 ± 46.79	112.33 ± 18.38	39.95 ± 4.80	165.73 ± 43.00	114.71 ± 18.96
*t-value*		− 0.400	0.221	0.272	− 0.475	− 3.268	− 0.302
*p-value*		0.690	0.826	0.786	0.636	0.002	0.763

### Comparison of oral nutritional supplementation adherence scores between the two groups of patients at postoperative day 7 and postoperative day 14

The adherence to oral nutritional supplementation was higher in the observation group than in the control group at 7 and 14 days postoperatively, and the differences were statistically significant (*t*-values of − 5.43 and − 5.35, respectively, *p* < 0.05), as shown in Table 4.

**Table 4:** Comparison of ONS adherence scores between the two groups of patients at 7 and 14 days postoperatively ($\overline{\mathrm{x} }$ ± s)

Group	ONS adherence score at 7 days postoperatively	ONS adherence score at 14 days postoperatively
Observation group	25.16 ± 5.34	25.95 ± 4.83
Control group	19.11 ± 4.86	20.57 ± 4.37
*t-value*	− 5.43	− 5.35
*p-value*	0.00	0.00

### Comparison of attainment of oral nutritional protein intake between two groups of patients 21 days after surgery

A comparison of the two groups’ attainment of oral nutritional protein intake at 21 days postoperatively showed that the attainment rate of the observation group was higher than that of the control group, and the difference was statistically significant (c2 value of 5.509, *p* < 0.05), as shown in Table 5.

**Table 5:** Comparison of the attainment rate of oral nutritional protein intake between the two groups of patients 21 days after surgery cases (%)

Group	Number of cases (*n*)	Attainment (*n*, %)	Non-attainment (*n*, %)	Difference 95%CI (%)	*χ*^2^ value	*p* value
Observation group	42	37 (88.1%)	5 (11.9%)	21.4 [4.13–38.73]	5.509	0.019
Control group	42	28 (66.7%)	14 (33.3%)			

## Discussion

### Nutrition education based on the goal attainment theory can effectively improve the nutritional status of colorectal cancer patients after surgery

Surgery is currently the main treatment for colorectal cancer, but surgical traumatic stress, altered gastrointestinal integrity or intestinal malabsorption, nausea from anesthesia, vomiting, loss of appetite, and changes in postoperative dietary patterns requiring a gradual transition from clear to fluid to semifluid can all lead to postoperative weight loss and the risk of malnutrition [21]. Malnutrition not only disrupts the physiological and immune functions of the body’s tissues, but also increases the incidence of complications such as postoperative sensory anastomotic fistula in patients and affects clinical outcomes. Therefore, NRS-2002 nutritional risk screening and nutritional status assessment should be performed for colorectal cancer patients in the perioperative period to develop individualized nutritional support programs for patients. The ERAS expert consensus in colorectal surgery [6] recommends ONS as the best mode of perioperative nutritional support; early resumption of transoral feeding and early implementation of ONS can reduce the incidence of complications, especially infectious complications. Nutrition education should be enhanced in ONS implementation to facilitate the smooth implementation of ONS.

In this study, we improved the outcome of postoperative ONS nutrition therapy in colorectal cancer patients by implementing a nutrition education program based on the goal attainment theory. The differences in preoperative serum albumin, prealbumin, and hemoglobin values between the two groups were not statistically significant (*p* > 0.05), and the baseline information was comparable. After preoperative intestinal preparation and surgical stress, there were no significant changes in serum albumin and hemoglobin in both groups 1 day after surgery. However, the anterior albumin was more sensitive in both groups and decreased to different degrees. Through nutrition education based on the goal-attainment theory, helping patients to develop energy and protein intake goal-attainment plans and individualized nutrition education, patients in the observation group had better prealbumin levels (200.25 ± 53.25) than the control group (165.73 ± 43.00) by 7 days postoperatively, with a *t*-value of − 3.268 and a *p*-value of 0.002, and the difference was statistically significant (*p* < 0.05). Prealbumin is a plasma protein synthesized by hepatocytes with a short half-life (2 days), and changes in its concentration can reflect the protein synthesis function of the liver early and sensitively and can be used as a sensitive indicator for detecting malnutrition [22]. The nutrition education program based on the goal attainment theory identifies patients’ nutrition cognitive misconceptions through assessment to improve patients’ ONS nutrition cognition and nurse-patient participation to complete the goal attainment program together to improve the effectiveness of treatment with nutrition support.

### Nutrition education based on the goal attainment theory can improve patient adherence to ONS nutrition support therapy

The results of the study on the factors influencing the knowledge of nutrition among colorectal cancer patients [23, 24] showed that the knowledge rate of colorectal cancer patients was 60.0%, and elderly people (> 60 years old) and low education level were the risk factors for the knowledge rate of nutrition among colorectal cancer patients, while nutrition education was the protective factor. ONS is currently the preferred nutritional support regimen for perioperative colorectal cancer patients, but both domestic and international studies [25, 26] have shown that patient adherence to ONS is generally low. Factors affecting ONS compliance include lack of knowledge about ONS, taste of ONS preparations, patients’ own conditions, intestinal tolerance, and environment [27]. Relevant qualitative studies [28] have shown that most patients have inadequate knowledge of ONS and a lack of positive attitudes toward taking it. The expert consensus on clinical management practices to improve adherence to oral nutritional supplementation [29] pointed out that nutrition support groups play an important role in standardizing the implementation of clinical nutrition therapy, improving patients’ nutritional status and clinical outcomes, and saving healthcare expenditures. In this study, members of the nutrition support team, led by a nutrition specialist nurse, implemented nutrition education based on the goal attainment theory and performed the management and supervision of nutrition based on conventional nutrition education. Patients in the observation group had good compliance in terms of ONS perception, method of configuration, number of doses, timing of dosing, dose, and concentration. The ONS treatment adherence scores were better than those of the control group at 7 and 14 days postoperatively, with statistically significant differences (*p* < 0.05). The reason may be related to nutrition education based on the goal attainment theory, focusing on individualization and improving cognition based on evaluation of patients’ behavioral feedback, continued follow-up, supervision, and repeated education. Through understanding patients’ perceptions and identifying nutritional misconceptions, the nutrition education program continuously follows up on patients’ ONS configuration and dosing methods to make patients and their families aware of the purpose and significance of proper ONS nutritional support and obtain more satisfactory results.

### Nutritional education based on the goal attainment theory can improve the attainment rate of patients’ oral nutritional intake

The occurrence of colon cancer is associated with poor dietary structure, excessive intake of pro-inflammatory diet, and dysbiosis of the intestinal flora [30, 31]. Foods rich in saturated fatty acids such as cream, cheese, fatty meats, and lard; buttered red meats, sausages, and cured meats; and carbohydrate foods with high glycemic index have pro-inflammatory effects, which can cause insulin resistance, promote excessive proliferation of colorectal epithelial cells and increase the risk of colorectal cancer [32, 33]. Fresh green vegetables, fruits, whole grains, sea fish, carotenoids, and dietary fiber have anti-inflammatory components with low dietary inflammatory index (DII), which can improve inflammation and oxidative stress in the intestine, maintain stable intestinal flora, and reduce the risk of colorectal cancer [34].

The diet of colorectal cancer patients should follow the principles of high protein, low fat, low fiber, low lactose, light and easy to digest, and balanced diet [35, 36], and the adult oral nutritional supplementation guidelines [8] recommend the ONS dose of diet plus ONS to reach the recommended daily energy and protein needs of the body, or the daily ONS to obtain at least 400–600 kcal in addition to the daily diet; the nutritional support guidelines for oncology patients [20] recommend that patients have a target protein requirement of 1.0–2.0 g/kg day, with a minimum requirement of 1.0 g/kg day. Patients’ dietary and ONS intake was recorded through homemade dietary and oral nutritional supplementation record forms, and the amount of protein consumed in the patient’s diet and the amount of protein consumed in ONS were calculated using the Mint Dietitian APP, and the minimum requirement of 1.0 g/kg day was reached, which was considered to be attained, and the forms were placed at the bedside during hospitalization and supervised by members of the nutrition team. After discharge from the hospital, patients’ diets and ONS intake were recorded on a form by the nurse through telephone follow-up. Since the change in dietary habits takes time and the family members who provide the meals are also related, this study focused on comparing the attainment rate of protein intake at 21 days postoperatively between the two groups of patients. The results of this study showed that patients in the goal attainment theory-based nutritional education group had better attainment rates of oral nutritional intake at 21 days postoperatively than the control group, with a rate of 88.1% for the observation group and 66.7% for the control group, with a chi-square value of 5.509 and a *p*-value of 0.019, and the difference was statistically significant (*p* < 0.05).

## Conclusion

In conclusion, nurses should adequately investigate and evaluate patients before conducting nutrition education for colorectal cancer patients and help patients change poor dietary habits and misconceptions about nutrition. In the process of nutrition education, nurses should make a comprehensive judgment of the patient’s nutritional status and combine their own dietary habits to provide personalized nutritional guidance to patients and family members on diet and help them gradually reach the daily target energy and protein requirements, and the part of insufficient dietary intake is supplemented by ONS. The nutrition education process should function as a nutrition support team led by specialist nurses to play the role of nutritional assessment, education, supervision, and management in patient nutrition support, thus improving the postoperative nutritional status of colorectal cancer patients. However, there are certain limitations in this study. First, the data on nutritional indicators were collected only from serum albumin, prealbumin, and hemoglobin values during the patients’ hospitalization, and no data on the patients’ long-term nutritional indicators were collected after discharge; second, there was no statistical difference in the baseline data of this study between the two groups in terms of cultural awareness level and surgical modality, but the patients’ diet, ONS intake compliance, and attainment during the intervention were related to the primary caregiver’s cognition, caregiving ability, the financial ability, and environment, which had some confounding factors. Finally, the sample size of this study was relatively small, and further expansion of the sample size is needed for the study.

## Data Availability

All data generated or analyzed during this study are included in this published article.
